# Left and right vagus nerve stimulation: historical perspectives, clinical efficacy, and future directions

**DOI:** 10.3389/fnhum.2025.1609654

**Published:** 2025-07-29

**Authors:** Birendra Sharma, Krysten A. Jones, Robert M. Lober, Candice N. Hatcher-Solis

**Affiliations:** ^1^Cognitive Neuroscience, 711th Human Performance Wing, Air Force Research Laboratory, Wright-Patterson AFB, OH, United States; ^2^Oak Ridge Institute for Science and Education, Oak Ridge, TN, United States; ^3^AV, Inc., Dayton, OH, United States; ^4^Department of Neurosurgery, Dayton Children's Hospital, Dayton, OH, United States

**Keywords:** left VNS, right VNS, clinical efficacy, epilepsy, cardiac function, depression, cognition

## Abstract

Neuromodulation has profoundly transformed medical science, offering new treatments for various neurological conditions. Stimulation techniques that target the brain, spinal cord, trigeminal nerve, and vagus nerve (VN) use electrical impulses to modulate neural functions. Among these, vagus nerve stimulation (VNS) is distinguished for its use to stimulate the VN to modulate neural functions. VNS shows promising applications across a wide range of neurological conditions, exemplifying the ongoing evolution of neuromodulation. As VNS continues to prove its efficacy, an important consideration in its application arises over the optimal VN stimulation site due to the bilateral nature of the VN. This review highlights the need for comparative studies of left VNS (L-VNS) and right VNS (R-VNS) to enhance our understanding of neurophysiology. The advantages and limitations of stimulation to the left VN or right VN are examined to potentially lead to more personalized and effective treatment strategies.

## 1 Introduction

The vagus nerve (VN), or cranial nerve X, coordinates a wide range of functions and is composed of approximately 80% sensory (afferent) and 20% motor (efferent) fibers (Bonaz et al., 2021). Sensory fibers from the left and right branches of the VN converge at the nucleus of the solitary tract (NTS) within the medulla oblongata, forming a crucial neural pathway to the brain (Jean, 1991). The NTS forms a direct, monosynaptic connection with multiple brain regions, including the amygdala, hippocampus, thalamus, hypothalamus, and locus coeruleus (LC). The LC further projects to these and other regions, contributing to the regulation of emotional and cognitive functions, neuroendocrine signaling, and central control of immune and barrier functions (Sawchenko, 1983; Barone et al., 1979; Sands et al., 2000; Sharma et al., 2010; Cottingham and Wang, 2012; Lopez et al., 2012; Verner et al., 2023; Olsen et al., 2023; Gargus et al., 2025). Building on these projections, the LC also connects to the basal forebrain, prefrontal cortex, midbrain, and dorsal raphe nucleus which influence alertness, arousal, attention, decision-making, mood, and behavior through dopaminergic and serotonergic pathways (Dorr and Debonnel, 2006; Peña et al., 2014; Collins et al., 2021).

Given the breadth of this neural connectivity, researchers have long been interested in how external modulation of the VN might influence central processes. This interest is not new; the clinical potential of modulating this pathway through vagus nerve stimulation (VNS) has been explored for over a century. The earliest therapeutic efforts date back to James Corning in the late 19th century, who attempted to suppress seizures via carotid artery compression and later incorporated vagal stimulation to enhance efficacy (Lanska, 2002). Although limited by side effects such as bradycardia and syncope, his work laid the foundation for the therapeutic use of VNS. Experimental animal models in the 1950s and pivotal canine studies by Zabara in the 1980s demonstrated the anticonvulsant potential of VNS (Yuan and Silberstein, 2016; Capilupi et al., 2020), culminating in the first human implantation in 1988 and subsequent FDA approval of left-sided VNS (L-VNS) for refractory epilepsy in 1997 (Penry and Dean, 1990; Ben-Menachem et al., 1994; Murphy et al., 1995).

Today, VNS can be delivered through invasive (iVNS) or non-invasive (nVNS) approaches. iVNS requires surgical implantation of a pulse generator and electrode cuff around the cervical VN, while nVNS uses surface electrodes applied to the ear (taVNS) or neck (tcVNS) (Ben-Menachem et al., 2015; Yap et al., 2020; Seitz et al., 2022). The efficacy of VNS is well-established in treatment-resistant epilepsy and depression, stroke, migraine, and most recent studies suggest benefits for cognitive performance and mood regulation even in healthy populations (DeGiorgio et al., 2000; Rush et al., 2005; Jacobs et al., 2015).

Despite these therapeutic advances, clinical research has historically favored L-VNS in part due to concerns surrounding the cardiac safety of right-sided VNS (R-VNS). These concerns stem from key anatomical differences between the left and right VN, which follow distinct trajectories and interact differently with the cardiac conduction system. The left VN traverses between the subclavian and carotid arteries and primarily innervates the atrioventricular (AV) node, a pathway generally considered safer for VNS. In contrast, the right VN courses near the sinoatrial (SA) node raising concerns about its potential to induce bradyarrhythmia (Erman et al., 2009; Kenny and Bordoni, 2022; Olshansky et al., 2008; Hammer et al., 2015; Capilupi et al., 2020; Ottaviani et al., 2022).

This anatomical difference contributed to early reports, predating and following FDA approval, describing bradycardia, asystole, and conduction abnormalities during L-VNS implantation (Sarnoff et al., 1960; Woodbury and Woodbury, 1990; Asconapé et al., 1999; Cantarín-Extremera et al., 2016; Lewis et al., 2001; Razmara et al., 2022). Despite efforts to optimize stimulation parameters, rare but persistent cardiovascular effects continue to be documented, raising critical questions about both L-VNS and R-VNS safety profiles. These historical, anatomical, and clinical considerations have contributed to a longstanding bias toward L-VNS in research and clinical practice. Yet, emerging studies now challenge this paradigm, suggesting R-VNS may be a viable and potentially advantageous approach for selected indications (McGregor et al., 2005; Navas et al., 2010; Premchand et al., 2016; Brougher et al., 2021). This review examines the evolving evidence base for L-VNS and R-VNS, emphasizing the need to re-examine assumptions about laterality to guide future research and clinical use.

## 2 Clinical applications of L-VNS and R-VNS

VNS has been applied in a range of clinical conditions, including epilepsy, depression, stroke rehabilitation, migraine, and cluster headaches. However, most of these lack evidence comparing the effects of L-VNS and R-VNS. Epilepsy and heart failure were selected for this review because both provide direct data relevant to stimulation laterality. Depression was included due to its long-standing clinical use with L-VNS and the absence of laterality-specific data, which remains unaddressed despite decades of therapeutic application. Cognition was included based on emerging studies that investigate both L-VNS and R-VNS effects in preclinical efforts. Summarized findings for each condition are presented in Tables 1–4, with R-VNS data included where applicable.

**Table 1:** Outcomes of L-VNS and R-VNS for epilepsy treatment in human patients.

Type of VNS	Model	Outcome(s)	Reference
L-VNS			
iVNS	Pediatric patients with medical refractory epilepsy	68% clinical response rate in children	Patwardhan et al. (2000)
iVNS	Adult patients with epilepsy	46% decrease in seizure frequency (3 months)	Englot et al. (2011)
iVNS	Adult patients with epilepsy	50% decrease in seizure frequency	García-Navarrete et al. (2013)
iVNS	Adult patients with epilepsy	63% long-term response rate (2 years)	Englot et al. (2015)
iVNS	Pediatric patients with primary generalized epilepsy	64% improvement in seizure frequency (1 year)	Welch et al. (2018)
iVNS	Adult patients with epilepsy	90% decrease in seizure frequency	Batson et al. (2022)
R-VNS			
iVNS	Pediatric patient with epilepsy	Seizure-free for over 2 years with minor respiratory events	McGregor et al. (2005)
iVNS	Medically refractory epilepsy	Seizure suppression, minor cardiac symptoms	Spuck et al. (2008)
iVNS	Adult patients with epilepsy	50 and 90% seizure reduction	Navas et al. (2010)
iVNS	Adult patients with epilepsy	Marked seizure reduction without cardiac side effects	Galbarriatu et al. (2015)

**Table 2:** Effects of L-VNS and R-VNS on heart failure management in human patients.

Type of VNS	Model	Outcome(s)	Reference
L-VNS			
iVNS	Patients with heart failure	Improve absolute LVEF by 4.5% and six-minute walk distance by 56 meters after 6 months	Premchand et al. (2016)
R-VNS			
iVNS	Patients with heart failure	Improvements in NYHA class and left ventricular function	Schwartz et al. (2008)
iVNS	Patients with heart failure	No significant impact on remodeling but improved quality of life	Zannad et al. (2015)
iVNS	Patients with heart failure	Improved quality of life	Gold et al. (2016)
iVNS	Patients with heart failure	Well-tolerated with maintained improvements (no significant differences when compared with L-VNS)	Premchand et al. (2016)
taVNS	Patients with heart failure	Reduced inflammatory cytokines and improved quality of life	Stavrakis et al. (2022)

**Table 3:** Results of L-VNS in treating depression and PTSD in human patients.

Type of VNS	Model	Outcome(s)	Reference
L-VNS			
iVNS	Patients with treatment-resistant depression	30% positive response	Rush et al. (2000)
iVNS	Patients with chronic or recurrent major depression	Progressive response and remission rates	Nahas et al. (2005); Schlaepfer et al. (2008)
iVNS	Patients with depression	Significant reductions in BDI scores	Cristancho et al. (2011)
iVNS	Patients with depression	20% response during the acute phase	Aaronson et al. (2013)
tcVNS	Patients with PTSD	Reversing neurobiological changes	Wittbrodt et al. (2021)
R-VNS			
No instances of R-VNS for applications in depression found at time of literature search			

**Table 4:** Impacts of L-VNS and R-VNS on cognitive function in human subjects.

Type of VNS	Model	Outcome(s)	Reference
L-VNS			
iVNS	Patients with epilepsy	Improved word recognition memory	Clark et al. (1999)
iVNS	Patients with Alzheimer’s disease	Positive effect on cognition	Sjögren et al. (2002)
iVNS	Patients with epilepsy	Enhancements in figural recognition tasks	Helmstaedter et al. (2001)
iVNS	Patients with epilepsy	Improved verbal memory retention	Ghacibeh et al. (2006)
tcVNS	Older adults	Increased associative memory performance	Jacobs et al. (2015)
taVNS	Patients with epilepsy	Reduced errors in working memory tasks	Sun et al. (2017)
tcVNS	Healthy adults	Increase performance in matrix reasoning tasks and fewer false negative errors in recognition tasks	Klaming et al. (2022)
R-VNS			
R-tVNS +L- tVNS	Sleep-deprived healthy adults	Enhancement in multitasking, arousal, and reduced fatigue-induced cognitive decline	McIntire et al. (2021)

### 2.1 L-VNS and epilepsy

Drug-resistant epilepsy is one of the primary conditions treated with iVNS, which received FDA approval in 1997, and extensive clinical research has shown the effectiveness of iVNS in reducing seizure frequency in patients with drug-resistant epilepsy (DeGiorgio et al., 2000). L-VNS has been used to treat epilepsy in both pediatric and adult populations. In pediatric patients with medically refractory epilepsy, particularly those with atonic seizures, iVNS had a 68% clinical response rate (Patwardhan et al., 2000). Long-term studies support the effectiveness of iVNS in children with partial refractory epilepsy (Rychlicki et al., 2006) showing seizure reductions over 2 years. Additionally, a one-year follow-up study in pediatric patients with primary generalized epilepsy reported a 64% reduction in seizure frequency (Welch et al., 2018).

In adult patients, multiple cases have demonstrated iVNS can significantly reduce seizure frequency by 50 to 90% (García-Navarrete et al., 2013; Batson et al., 2022). Studies have also shown a progressive increase in iVNS clinical benefits over time, with a 46 and 62% decrease in seizure frequency after 3 months or 2 years, respectively (Englot et al., 2011). Long-term response rates in larger cohorts indicated a 63% reduction in seizure frequency after 2 years of treatment (Englot et al., 2015).

The mechanism of how L-VNS treats epilepsy involves multiple potential pathways. In humans, acute L-VNS enhances thalamic blood flow, with increased thalamic activity tied to fewer seizures, suggesting thalamic synaptic mediation (Henry et al., 1999). L-VNS also promotes EEG desynchronization particularly in theta and gamma bands, disrupting pathological neural synchrony (Sangare et al., 2020). A recent review of the literature suggests L-VNS modulates epileptic networks through a cascade beginning in the brainstem and extending to limbic and cortical regions, reducing hyperconnectivity and cortical excitability (Carron et al., 2023).

### 2.2 R-VNS and epilepsy

Several studies have explored the use of R-VNS as an alternative to L-VNS for treating epilepsy. An observational report described three children who benefited from L-VNS, but L-VNS was discontinued early due to infections, and R-VNS was applied. One child became seizure-free for over 2 years with R-VNS and experienced no postoperative cardiac side effects, although minor respiratory events, which are also common with L-VNS, were noted. Another child saw an improvement in seizure control, albeit less dramatic than with L-VNS. The third child experienced a cessation of generalized tonic–clonic seizures, with only transient respiratory issues after swimming (McGregor et al., 2005).

In another case, a 16-year-old boy with medically refractory psychomotor seizures initially responded to L-VNS but required removal of the pulse generator due to a deep wound infection which prevented left-sided surgery. R-VNS was therefore implanted, leading to seizure suppression, although with a delayed onset compared to L-VNS. However, stimulation of the right VN induced minor cardiac symptoms, necessitating ECG-guided placement and adjustment of the device to manage potential adverse effects on cardiac function (Spuck et al., 2008).

Two adult patients additionally underwent R-VNS following complications with L-VNS implantation. While one patient experienced significant improvements in seizure reduction with L-VNS, the stimulation device was removed due to mechanical malfunction and device reimplantation in the left VN was deemed too risky. The device was therefore placed in the right VN, and the subsequent R-VNS yielded a 95% seizure reduction without cardiac side effects. The utility of L-VNS for the second patient was halted due to significant bleeding, prompting a switch to R-VNS, which led to a 50% control of seizures without any cardiac complications (Navas et al., 2010).

In a medical series reported by Galbarriatu et al. (2015), one patient underwent R-VNS, which resulted in a seizure burden reduction comparable to that achieved with L-VNS. There were no significant cardiorespiratory events reported either in the immediate post-operative period or in the longer follow-up. Collectively, these cases highlight R-VNS as a viable alternative when L-VNS is unsuitable due to individual conditions or complications, underscoring the potential for off-label VNS applications to provide significant clinical benefits for epilepsy.

### 2.3 L-VNS and cardiac regulation

While the focus has often been to stimulate the R-VNS due to its innervation to the SA node to stimulate heart activity, research has shown that L-VNS also provides significant cardiovascular benefits, in the management of heart failure.

In humans, the ANTHEM-HF study demonstrated that chronic iVNS in heart failure patients with reduced ejection fraction was safe, feasible, and associated with significant clinical benefits (Premchand et al., 2016). Sixty patients with symptomatic heart failure (LVEF ≤40%, NYHA Class II–III) were enrolled and randomized to receive either L-VNS or R-VNS. After six months of therapy, L-VNS led to an average increase in left ventricular ejection fraction from 32.0 to 37.2%. Both groups showed gains in six-minute walk distance, with the left-sided group improving by 58.4 meters. Heart rate variability also improved in both arms, and there were no device-related serious adverse events. There were no statistically significant differences in efficacy between L-VNS and R-VNS, and the data were pooled for extended analysis. However, in the larger ANTHEM-HFrEF trial of over 500 patients, iVNS did not significantly improve primary clinical endpoints, and outcomes were not reported separately by stimulation side, limiting conclusions about laterality (Konstam et al., 2019, 2024).

### 2.4 R-VNS and cardiac regulation

Clinical studies investigating R-VNS for cardiac regulation have yielded mixed results. The first human trial using the CardioFit system showed improvements in New York Heart Association class, quality of life, and left ventricular end-systolic volume in eight patients with heart failure (Schwartz et al., 2008). The NECTAR-HF trial found R-VNS did not significantly impact cardiac remodeling and functional capacity but did improve quality of life measures (Zannad et al., 2015). The INOVATE-HF trial reported that while R-VNS improved quality of life and functional class, it did not significantly reduce the rate of death or heart failure events in patients (Gold et al., 2016).

In the ANTHEM-HF patients receiving R-VNS experienced a mean increase in left ventricular ejection fraction from 32.9 to 39.0%, along with a 70.5-meter improvement in six-minute walk distance over 6 months. Heart rate variability improved, and importantly, device-related adverse events were significantly lower in the right-sided group, with a 4:1 ratio favoring R-VNS. The study indicated that autonomic regulation therapy via R-VNS was well-tolerated and maintained improvements in heart function and symptoms over a 12-month period (Premchand et al., 2016). However, the subsequent ANTHEM-HFrEF trial did not replicate these benefits and failed to show significant improvement in primary clinical outcomes (Konstam et al., 2019, 2024). Most recently, a sham-controlled, double-blind, randomized clinical trial demonstrated significant improvements in global longitudinal strain, inflammatory cytokines, and quality of life in patients with heart failure with preserved ejection fraction through taVNS (Stavrakis et al., 2022). Importantly, no device-related side effects were observed. These collective findings suggest that while R-VNS shows promise as a treatment for heart failure, further research is necessary to fully understand its efficacy and optimize its clinical application in humans.

### 2.5 L-VNS and depression

L-VNS is an effective FDA-approved treatment for depression in patients who do not respond to conventional antidepressants. Initial studies by Rush et al. (2000) demonstrated that over 30% of participants experienced a positive response on the Hamilton Depression Rating Scale (HDRS) following L-VNS. A subsequent year-long clinical trial confirmed significant and sustained mood improvements (Rush et al., 2005). Additional longitudinal studies have also shown progressively improving response and remission rates in patients with chronic or recurrent major depressive episodes (Nahas et al., 2005; Schlaepfer et al., 2008). Significant reductions in additional clinical depression indexes, including Beck Depression Inventory (BDI) scores, have also been observed over time after iVNS administration with scores decreasing from a baseline mean of 37.8 to 24.6 at 12 months, along with a 28.6% response rate and 7.1% remission at 1 year (Cristancho et al., 2011).

Investigations into optimal iVNS dosages have shown 20% of participants responded positively during the acute phase, as assessed primarily using HDRS (Aaronson et al., 2013). Long-term benefits of iVNS in treatment-resistant depression were further substantiated with sustained positive outcomes on both HDRS and BDI scores in a five-year study (Aaronson et al., 2017). The durability of therapeutic effects of L-VNS was further supported by studies showing enhanced clinical outcomes and improved quality of life for patients over extended periods (Conway et al., 2018; Kumar et al., 2019). Additionally, explorations into left tcVNS have shown promising outcomes, with the potential for reversing neurobiological changes associated with PTSD (Wittbrodt et al., 2021).

One potential mechanism behind the antidepressant effects of VNS may involve the upregulation of brain derived neurotrophic factor (BDNF) through noradrenergic signaling from the locus coeruleus, a mechanism shared with traditional antidepressants (Saarelainen et al., 2003; Follesa et al., 2007; Yang et al., 2020). Beyond its influence on BDNF, VNS also modulates several neurotransmitter systems that are critical for mood regulation. L-VNS has been shown to enhance serotonergic output from the raphe nuclei, increase dopaminergic activity in the ventral tegmental area, and promote neuroplastic remodeling in the hippocampus and cortex. It further reduces GABAergic inhibition and alters activity in key brain regions such as the insular cortex, anterior cingulate, and orbitofrontal cortex. These combined effects are thought to restore functional connectivity within mood-related networks and may underlie the antidepressant properties of VNS (Conway and Xiong, 2018).

### 2.6 L-VNS and cognition

L-VNS has shown promise in enhancing cognitive function in healthy individuals and patients with epilepsy. Following L-VNS administration, significant improvements in word recognition memory, figural recognition tasks, and verbal memory retention have been observed (Clark et al., 1999; Helmstaedter et al., 2001; Ghacibeh et al., 2006). Additionally, taVNS has also been shown to effectively reduce errors in working memory tasks among epileptic adults when administered before and during learning (Sun et al., 2017). In patients with Alzheimer’s disease, iVNS improved Alzheimer’s disease assessment scale-cognitive subscale and mini-mental state examination scores over 3 and 6 months, potentially enhancing cognitive functions and memory retention by decreasing tau protein accumulation and improving cerebral blood flow (Sjögren et al., 2002; Merrill et al., 2006).

Recent studies involving healthy populations have also demonstrated the potential of non-invasive L-VNS for cognitive enhancement. tcVNS increased associative memory performance in older adults when applied during and after learning (Jacobs et al., 2015). Additionally, tcVNS significantly increased performance in matrix reasoning tasks and reduced false negative errors in recognition tasks when administered before learning in healthy humans (Klaming et al., 2022). A recent review of the literature indicates VNS enhances memory by activating the NTS and subsequently the LC, increasing norepinephrine release and inducing hippocampal synaptic plasticity (Olsen et al., 2023). Collectively, these studies underscore L-VNS as a promising intervention for cognitive enhancement, highlighting its potential for broader applications in neurological therapy.

### 2.7 R-VNS and cognition

While research on R-VNS and cognition is less extensive than L-VNS, a notable study by McIntire et al. (2021) bridges this gap by applying tcVNS to the left and right sides of the neck in healthy military personnel during 34 h of sleep deprivation. Bilateral tcVNS enhanced multitasking and arousal performance, with a 5% throughput decline compared to 15% in the sham group, alongside reduced fatigue ratings. This suggests that combined L-VNS and R-VNS may offer robust cognitive benefits, potentially via synergistic activation of the locus coeruleus-norepinephrine system, warranting further exploration into the specific effects of R-VNS.

## 3 Discussion

The evolution of VNS has demonstrated significant therapeutic potential, with established clinical use in epilepsy and depression, and growing investigational interest in heart failure and cognitive disorders. Understanding the broader impact and future potential of this therapy requires examining key developments, clinical applications, and the comparative efficacy of L-VNS and R-VNS. Cross-species and clinical studies suggest that both left and right stimulation can be administered safely and effectively, with outcomes influenced more by anatomical targeting, stimulation parameters, and individual variability than by laterality alone.

### 3.1 Cardiac safety and anatomical consideration in R-VNS and L-VNS

Recent human studies have shown that R-VNS does not inherently pose a greater risk of cardiac side effects compared to L-VNS in epilepsy treatment. This is in contrast to outcomes in animal models and are likely due to significant anatomical differences. For instance, in dogs, pronounced cardiac effects like bradycardia or asystole may arise when stimulation occurs proximally because of more distally originating cervical cardiac nerves (Schuessler et al., 1986). Individual anatomical variations in humans can inadvertently stimulate cardiac fibers, causing bradycardia during both R-VNS and L-VNS; however, these effects are infrequent and often detected during intraoperative testing (Cantarín-Extremera et al., 2016). Cardiac side effects during VNS in humans can also result from improper electrode placement, stimulation parameters, device malfunctions, and polarity reversal. This may lead to erratic stimulation intensities or enhanced cardiac responses (Frei and Osorio, 2001; Amark et al., 2007; Spuck et al., 2008; Iriarte et al., 2009). Furthermore, rare respiratory side effects like dyspnea and worsening asthma observed with both R-VNS and L-VNS may result from the stimulation of capsaicin-sensitive afferent fibers (Undem et al., 1990; McGregor et al., 2005). A recent multicenter review further supports these findings, reporting slightly lower tolerability in epilepsy patients receiving R-VNS, with adverse effects including dyspnea, hoarseness, and sleep-related symptoms (Zanello et al., 2025). Furthermore, MRI studies suggest that the cardiac effects observed may result from long-term VNS-induced changes in the NTS and its projections to other brain nuclei, impacting higher autonomic functions (Narayanan et al., 2002; Amark et al., 2007). These findings emphasize that both R-VNS and L-VNS can be administered safely under controlled conditions with careful attention to technical and anatomical considerations.

### 3.2 Comparative efficacy and safety of R-VNS and L-VNS in heart failure

Initial concerns about potential cardiac complications from R-VNS have not been substantiated in clinical trials and animal studies. For instance, the ANTHEM-HF trial demonstrated R-VNS maintains a good safety profile and improves the quality of life in patients with heart failure, showing no significant differences in efficacy between R-VNS and L-VNS (Premchand et al., 2016). Importantly, device-related adverse events were lower in the right-sided group, suggesting favorable tolerability. Further research into the effects of R-VNS and L-VNS on cardiac repolarization and hemodynamics in a porcine model found both methods equally increased action potential duration (APD) across various heart regions. There was no significant laterality in their effects with greater prolongation of APD at the heart’s apex than at the base and similar impacts on the anterior, posterior, and lateral walls of both ventricles (Yamakawa et al., 2014). Hemodynamic responses, including left ventricular end-systolic pressure and the rate of pressure development, showed no differences between L-VNS and R-VNS, indicating a lack of functional laterality. Additionally, studies with hypertensive rats have shown L-VNS induced more pronounced bradycardia, hypotension, and tachypnea compared to R-VNS (Stauss, 2017). These differences in cardiovascular and respiratory responses were influenced by stimulation parameters: lower frequencies and shorter pulse durations activated larger A-fibers, while longer pulse durations recruited smaller B-fibers. These findings collectively suggest that R-VNS and L-VNS can be safely and effectively used in heart failure treatment, emphasizing the importance of careful attention to stimulation parameters to optimize outcomes.

### 3.3 Enhanced therapeutic potential of R-VNS in dopaminergic and noradrenergic activation

Recent findings in rodents have revealed a compelling advantage in targeting the right cervical VN, especially in activating the dopaminergic midbrain and noradrenergic neurons in the LC. A study in Long-Evans rats tested the differential effects of VNS when self-administered to the right or left cervical VN, providing the first evidence that R-VNS reinforces learned behaviors, which was absent with L-VNS (Brougher et al., 2021). The enhanced behavior correlated with a significant rise in c-Fos expression within tyrosine hydroxylase-positive (TH+) neuronal populations in the ventral tegmental area and substantia nigra pars compacta, suggesting R-VNS may enhance therapeutic outcomes for diseases like Parkinson’s by more effectively engaging the dopaminergic pathways. Additionally, R-VNS led to a higher percentage of TH + cells in the LC, corroborating previous research indicating the right cervical branch of the VN possesses significantly more TH + nerve fibers than the left cervical branch (Verlinden et al., 2016). The increased number of TH + cells is associated with greater availability of catecholamines, such as dopamine, norepinephrine, and epinephrine, as well as elevated levels of neurotrophic factors like BDNF (Weihe et al., 2006; Farrand et al., 2020). While L-VNS has been shown to exert its beneficial effects by activating noradrenergic neurons and promoting the release of norepinephrine and BDNF (Bonaz et al., 2019; Berger et al., 2021; Olsen et al., 2023), the greater potential for norepinephrine and BDNF production with R-VNS suggests it could offer similar or even superior clinical benefits. However, how these findings translate to humans has yet to be determined.

## 4 Conclusion

L-VNS has traditionally been favored due to concerns about cardiac side effects from R-VNS. However, recent studies suggest R-VNS may not pose significantly greater cardiac risks and could offer unique therapeutic benefits. Current studies on R-VNS have primarily focused on its cardiac and anticonvulsant effects, leaving a gap in understanding its impact on patient populations suffering from depression, which have been traditionally treated with L-VNS. Addressing this gap is crucial, as some patients experience complications with the left side of the body or insufficient responses to L-VNS. Cognitive outcomes have also begun to attract attention, with limited evidence comparing both stimulation sides. Further research is needed to explore the potential of R-VNS in these areas, optimizing stimulation parameters, and understanding its broader mechanisms. By expanding research efforts, the scientific community can better assess the full therapeutic potential of R-VNS, potentially offering improved treatment options for a wider range of conditions leading to improved patient outcomes.
